# Shifts in endocrine homeostasis and preventive hormone replacement therapy: extending the Women’s Health Initiative globally

**DOI:** 10.1186/s41256-016-0009-4

**Published:** 2016-07-30

**Authors:** Jacob D. Ball, Xinguang Chen

**Affiliations:** Department of Epidemiology, College of Public Health and Health Professions & College of Medicine, University of Florida, 2004 Mowry Road, CTRB #4228, Gainesville, FL 32610 USA

**Keywords:** Hormone replacement therapy, Menopause, Dynamic endocrine changes, Women’s health

## Abstract

**Background:**

Reducing disease risk for women after menopause is global health issue. A major portion of the Women’s Health Initiative (WHI) consisted of two clinical trials involving 161,809 post-menopausal women aged 50–79 that tested the effect of hormone replacement therapy (HRT) on reducing cardiovascular disease and other secondary outcomes. Previous analyses of the data reveal that HRT should not be recommended for post-menopausal women, but show potential benefits for younger women. Thus, there may be a critical period just prior to or during the early stages of menopause where HRT could be both safe and beneficial.

**Main body:**

Menopause marks the beginning of a process of non-reversible reduction in estrogen by which estrogen levels decline progressively, followed by a reduction in estrogen receptors. This results in periods of hormone-receptor imbalances, exacerbating the effects of lower serum estrogen and is considered the primarily endocrinal source of menopause symptoms. Eventually a hormone-receptor balance is achieved at a lower level.

Here, we purport that the negative outcomes from WHI trials were primarily due to the fact preventive HRT was initiated in women who had already achieved hormone-receptor equilibrium at lower hormonal levels.

**Conclusion:**

We argue for further HRT clinical trials in women at varying stages of menopause, including pre-menopause and early menopause, and in women from different countries. Variation across countries and subgroups in how women experience menopause and perceive menopause symptoms suggest that biocultural differences should be considered in both study design and measurement approaches to test the effectiveness of HRT. Particularly, we recommend longitudinal studies to assess changes in hormonal level over time, and to detect the “most effective period” for HRT to reduce health risk for women going through the whole menopause period.

## Background

Reducing disease risk for women after menopause is global health issue. A group of leading scientists from a number of universities in the United States launched the program Women’s Health Initiative (WHI) in 1991 with funding primarily from the U.S. National Institutes of Health. A major portion of WHI consists of two clinical trials involving 161,809 post-menopausal women aged 50–79 from 40 clinics. The two trials tested the effect of hormone replacement therapy (HRT) on reducing cardiovascular disease (CVD), with invasive breast cancer (IBC), hip fracture, stroke, and more as secondary outcomes (e.g. [4, 9, 10]). The trials were halted due to observed consistent increases in CVD and IBC risks [9].

More recently, Manson and colleagues performed an age-stratified analysis of the WHI data, including additional years of follow-up and concluded that, similar to previous analyses, HRT should not be recommended for chronic disease prevention in post-menopausal women [4, 5, 9]. However, Manson’s analysis reveals benefits to an estrogen-only regimen for younger women (e.g., those aged 50–59), suggesting there may be a critical period just prior to or during the early stages of menopause to initiate preventive HRT. Similar critical period hypotheses have been examined with respect to HRT and Alzheimer’s disease (e.g. [6, 7]), and with HRT and cognition and mood [2] with varying results.

These studies were conducted in post-menopausal Caucasian women in the United States, rather than women from multiple countries and ethnic backgrounds that are in pre- and early-menopause. We argue that the critical window theory should be further examined in geographically and culturally diverse women cohorts who in multiple stages of menopause and for other health outcomes in a global setting before dismissing HRT as dangerous in all postmenopausal women.

Menopause marks the beginning of a process of non-reversible reduction in estrogen. According to the [1], during the approximately one-year of menopausal transition, estrogen levels decline progressively, followed by a reduction in estrogen receptors. This results in periods of hormone-receptor imbalances, exacerbating the effects of lower serum estrogen and is considered the primarily endocrinal source of menopause symptoms. The subsequent alleviation of the menopause symptoms during the early menopause period (3–4 years) indicates a new, hormone-receptor equilibrium was reached at low hormonal levels, which is associated with an increased risk of CVD. The rationale of the WHI trials was to supply estrogen with an expectation to curb this risk of CVD [3].

The negative outcomes from WHI trials were primarily due to the fact preventive HRT was initiated in women who had already achieved hormone-receptor equilibrium at lower hormonal levels [5]. Hormonal therapy at this period will disturb the established balance. Endocrinologically, it would be more favorable if hormone therapy were to be initiated during the premenopausal transition period when the new hormone-receptor equilibrium at the low hormonal levels has not yet been established [1]. Appropriate levels of estrogen supplementation during this period would minimize periods of hormone-receptor imbalance and would thus allow women to proceed through the menopausal transition at a slower pace, lessening menopause symptoms and reducing other health risks, including cardiovascular disease [3].

## Conclusions

Furthermore, globally, women’s hormonal levels and the changes in those levels during menopause can vary widely between countries. Similarly, how women experience menopause and perceive menopause symptoms suggest that biocultural differences should be considered when selecting both study design and measurement approaches to test the effectiveness of HRT [8]. Particularly, we recommend longitudinal studies to assess changes in hormonal level over time, and to detect the “most effective period” for HRT to reduce health risk for women going through the whole menopause period.

There is significant need for additional longitudinal studies like the WHI in multiple international contexts to test this hypothesis by recruiting and following women in different countries based on their stage of menopause, and to tease out confounding from lifestyle, cultural and genetic variation. Through these studies, we could devise appropriate HRT dosage and timing recommendations based on existing knowledge of hormone and receptor decline following menopause using a time-since-menopause analysis [3], and design the trials to address the variable health concerns of the multiple populations that are represented. While it might be technically and practically challenging to identify and recruit women in menopausal transition stage for research purpose, the criterion for menopause transition established by [1] provide a solid foundation for developing valid measurement tools to address this challenge. Appropriately timed HRT has the potential to not only alleviate menopause symptoms, but also to reduce risk of CVD, IBC and other negative health outcomes across the globe.
